# Resveratrol Inhibits Pseudorabies Virus Replication by Targeting IE180 Protein

**DOI:** 10.3389/fmicb.2022.891978

**Published:** 2022-06-02

**Authors:** Xiangxiu Chen, Xu Song, Lixia Li, Yaqin Chen, Renyong Jia, Yuanfeng Zou, Hongping Wan, Ling Zhao, Huaqiao Tang, Cheng Lv, Xinghong Zhao, Zhongqiong Yin

**Affiliations:** ^1^Natural Medicine Research Center, College of Veterinary Medicine, Sichuan Agricultural University, Chengdu, China; ^2^Key Laboratory of Animal Disease and Human Health of Sichuan Province, Sichuan Agricultural University, Chengdu, China

**Keywords:** resveratrol, pseudorabies virus, IE180 protein, antiviral, molecular docking

## Abstract

Resveratrol is a natural polyphenolic product in red wine and peanuts and has many pharmacological activities in humans. Our previous studies showed that resveratrol has good antiviral activity against the pseudorabies virus (PRV). However, little is known about the antiviral mechanism of resveratrol against PRV. In this study, we found that resveratrol inhibited the nuclear localization of IE180 protein, which is an important step for activating early/late genes transcription. Interestingly, the results show that resveratrol inhibited the activity of IE180 protein by dual-luciferase assay. Furthermore, molecular docking analysis shows that resveratrol could bind to the Thr601, Ser603, and Pro606 of IE180 protein. Point mutation assay confirmed that resveratrol lost its inhibition activity against the mutant IE180 protein. The results demonstrate that resveratrol exerts its antiviral activity against PRV by targeting the Thr601/Ser603/Pro606 sites of IE180 protein and inhibiting the transcriptional activation activity of IE180 protein. This study provides a novel insight into the antiviral mechanism of resveratrol against herpes viruses.

## Introduction

*Herpesviridae* is a large family of enveloped-double-stranded DNA viruses, divided into three subfamilies, α, β, and γ, according to their genomic sequences and etiological characteristics (Kofahi et al., 2020). The subfamily α includes Herpes simplex virus 1 and 2 (HSV-1 and HSV-2), pseudorabies virus (PRV), and varicella-zoster virus (VZV) which are characterized by rapid replication (Xie et al., 2019). Interestingly, members of the *Herpesviridae* family share the same virion structure as the viral genome, the capsid, the interstitial protein layer, and the capsule membrane, respectively. Moreover, these viruses encode immediate-early regulatory proteins, which play an essential role in transactivating viral gene expression (Antinone and Smith, 2010; Lerma et al., 2020). Viral gene expression is tightly regulated in a cascading manner. Immediate early genes, early genes, and late genes are expressed successively. The immediate-early protein functions of these viruses are highly similar. Notably, HSV-1 has five immediate-early proteins, including ICP0, ICP4, ICP22, ICP27, and ICP47 (Huang et al., 2019). The pORF62, pORF63, and pORF10 are immediate-early regulatory proteins of ZVZ, while PRV has only one IE180 protein (Jiang et al., 2017).

The PRV is a swine pathogen and is characterized by sow reproductive failure, high piglet fatality rate, and latent infection of fattening pigs, inducing enormous economic losses around the world (Zhou et al., 2020). The PRV IE180 protein is the only immediate-early transcriptional activator of viral genes and plays a crucial role in translating viral genomes (Wu et al., 2014). The expression of E genes during infection is dependent on IE180 protein, while the expression of L genes is dependent on DNA replication. The IE180 gene is present in two copies in the genome, located in the IRS and TRS repeats (Guan et al., 2018). A nuclear localization signal was mapped to the positively charged region (amino acids 930–935) RRKRR. The role of IE180 as a potent transcriptional activator has been reported, and the transcription of IE180 did not require any viral protein synthesis (Huang and Wu, 2004; Lerma et al., 2016). Like most typical cellular activators, IE180 contains a separate domain for DNA-binding and another for trans-activation. IE180 is the only protein that activates downstream gene transcription, and IE180-null mutant induced failure of replication of their genome, synthesis of viral protein, and production of infection progeny (Ono et al., 1998). Therefore, the IE180 protein could be a valuable target for controlling PRV proliferation and latency establishment.

Resveratrol (3,5,4′-trihydroxystilbene, Res, Supplementary Figure S1) is a non-flavonoid polyphenol compound and wildly exists in plants and fruits, peanuts, and grapes, at an especially high concentration in the red wine (Thapa et al., 2019). Res has acquired increasing importance due to their antiviral, anti-inflammatory, antioxidant, and cardiovascular protection activities (Zhuang et al., 2019; Banez et al., 2020; Huang et al., 2020). Our previous studies confirmed that the effective concentration of 50% (EC_50_) of resveratrol against PRV was 3.92 μg/ml *in vitro* (Zhao et al., 2018). *In vivo* study also revealed that resveratrol can reduce the mortality of PRV-infected piglets and inhibit the replication of the virus in various organs (Zhao et al., 2017).

Our study defines Res binds to the IE180 protein, leading to inhibition of the translocation of IE180 from the cytoplasm to the nucleus, thus preventing the IE80 protein from activating downstream gene expressions of PRV. This study provides new insights into the prevention of herpesvirus infection by Res.

## Materials and Methods

### Chemical

Res was purchased from Solarbio Life Sciences Co., Ltd. (Beijing, China) with a purity of 98%. Res was dissolved in ethanol in the assays and then diluted by Dulbecco’s modified eagle medium (DMEM) to 15 μg/ml. The cells in the control group were treated with corresponding DMEM dilutions lacking Res.

### Cell Culture and Virus

The PRV (RA strain) was purchased from China Veterinary Culture Collection Center (Beijing, China). The swine kidney cell line PK-15 and HEK293T cell, obtained from China Center for Type Culture Collection (Wuhan, China), were propagated in DMEM supplemented with 10% fetal bovine serum (FBS), 100 U/ml penicillin, and 100 μg/ml streptomycin. The cells were cultured at 37°C in an atmosphere of 5% CO_2_.

### Plasmids Construction and Transfection

Full-length DNA of IE180 was sub-cloned into the pcDNA3.1 (+) vector (pIE180; Miaoling, Wuhan, China; Supplementary Figure S2). To construct luciferase reporter plasmid pGL3-TK, the DAN fragment of the TK promoter region was inserted into pGL3-Basic-Vector (Promega, United States; Supplementary Figure S3). All constructs were verified by DNA sequencing. The primer sequences are listed in Supplementary Table S1. One day prior to transfection, HEK293 cells (China Center for Type Culture Collection, Wuhan) were seeded in 24-well plates (2.5 × 10^6^ cells/well). Transfection of the plasmids was performed using the Lipofectamine™ 3,000 Transfection Reagent (Thermo Fisher Scientific) when cells reached 80% confluence cultured in 24-well plates. Transfection was performed according to the manufacturer’s protocol with 125 ng pcDNA3.1 (+)/pIE180/pIE180^Thr601Ala^/pIE180^Ser603Ala^/pIE180^Pro606Ala^ (Supplementary Figure S4) 325 ng pGL3-TK and 50 ng pRL-TK (Promega, United States) plasmids together. The 1 μl P3000™ and 0.75 μl Lipofectamine™ 3,000 were used for each well during transfection. Three biological replicates were performed for each sample. All the plasmids involved are shown in Supplementary Table S2.

### Real-Time PCR

According to the manufacturer’s instruction, total RNA from cells was extracted by the total RNA Kit П (R6934-01, OMEGA). Then, the RNA was immediately reverse-transcribed into cDNA using the iSCRIPT cDNA SYNTHESIS Kit (Thermo scientific, United States). Then, the RT-PCR was performed with iQ SYBR Green Supermix kit (Bio-Rad, United States). The reaction contained in a final volume of 10 μl: SYBR Green PCR Master Mix (5 μl), each primer set (1 μl), and purified cDNA (1 μl), DNase-free water (3 μl). The RT-PCR cycling was 3 min at 95°C, followed by 40 cycles of 95°C for 10 s (denaturation), 56°C for 30 s (annealing), and 72°C for 30 s (extension). Expression of β-actin was used to normalize the differences in total cDNA levels in the samples. Relative quantification was determined using Bio-Rad CFX96 Manager software by calculating 2^−ΔΔCt^. Primer sequences are provided in Supplementary Table S3.

### Western Blot Analysis

To analyze viral protein accumulation, PK-15 cells were infected with PRV (MOI = 5) for 1 h, followed by treatment with or without 15 μg/ml of Res at 4, 6, and 8 h, respectively. In the over-expression assay, the recombinant pIE180 was transfected into PK-15 cells for 24 h. Then, the PK-15 cells were infected with PRV (MOI = 5) for 1 h. The cells were then treated with or without Res (15 μg/ml) at various times (4, 6, and 8 h, respectively). The cells were collected and treated with lysate buffer (solarbio, China), and the concentration of total proteins was detected using a bicinchoninic acid assay (solarbio, China). The viral proteins were analyzed by western blotting as described previously (Nie et al., 2020). The PVDF membrane was imprinted with anti-IE180 (1:200; GenScript, China) and anti-β-actin antibodies (1:5000; Solarbio, China). After the Super Signal ECL reagent (Bio-Rad, United States) was added. The proteins were detected, and band intensities were quantified using Image J.

### Immunofluorescence Assay

PK-15 cells were propagated in a six-well plate (2.5 × 10^6^ cells/well) to ~90% containing glass coverslips and were infected with PRV (MOI = 5) in the absence or presence of Res (15 μg/ml). As described previously, an immunofluorescence assay was performed (Marey et al., 2020). Briefly, the coverslips were washed with PBS and filled with 4% fresh formaldehyde at room temperature for 15 min. 5% BSA was used to block at 37°C for 30 min. The coverslips were washed four times with PBS and incubated with anit-IE180 antibody (1:200) at 4°C overnight. After washing, a secondary antibody (1:1000, rabbit, Bioss, China) was added. After incubating at 37°C for 1 h, the coverslips were washed, and DAPI (Solarbio, Beijing, 1:5000) was added. Finally, the coverslips were washed and observed under a fluorescence microscope (Nikon, Japan).

### Molecular Docking

The molecular structure of Res was searched using ChemDraw 18.0, and the 3D ligand was saved in mol format. Hydrogenation, energy minimization, and structure optimization of ligands can be performed in Discovery Studio 2016 Client software. The structure of IE180 was assessed by X-ray structure, Ramachandran plot, and Profile-3D model in the Discovery Studio. At the same time, The PDB file chosen for the molecular docking-based virtual screening study was processed by removing water molecules, adding hydrogen atoms, and finally prepared by Discovery Studio 2016. Molecular docking was performed to identify binding affinity and interactions between synthesized compounds and receptors using Small Drug Discovery Suites package (Turkan et al., 2019). The protein-ligand complexes results were then analyzed.

### Mutant Generation

Based on the molecular docking results, the mutation of the binding sites of Res with IE180 was conducted to confirm the binding sites. The mutant plasmids (pIE180^Thr601Ala^/pIE180^Ser603Ala^/pIE180^Pro606Ala^) were generated using the primers list in Supplementary Table S4. According to the manufacturer’s instructions, the PCR reaction was carried out using TKs GfIex® DNA Polymerase (Takara, Japan). The thermocycler was programmed as follows: initial denaturation at 98°C for 10 min, followed by 30 cycles of 98°C for 10 s, annealing temperature of 60°C for 15 s, and 68°C for 1 min, and a final extension at 72°C for 10 min. The PCR products were analyzed by DNA agarose gel electrophoresis and purified using a Takara MiniBEST Agarose Gel DNA Extraction Kit Ver. 4.0 (Takara, Japan).

### Dual-Luciferase Reporter Assay

The HEK293T cells were seeded into a 24-well plate (~80% confluency) and were transfected with pcDNA3.1 (+)/pIE180/pIE180^Thr601Ala^/pIE180^Ser603Ala^/pIE180^Pro606Ala^ together with pGL3-TK and control pRL-TK vector for 24 h. Luciferase activity was measured using the Dual-Glo luciferase Assay System (Promega, E1910, United States) after Res treatment for 48 h. Briefly, the Dual-Glo luciferase substrate was added to each well. The firefly luminescence signal (FiLuc) was read after 10 min using the plate reader. Then, the Stop-Glo substrate was added. Following a second 10 min incubation, the Renilla luciferase signal (hRLuc) was recorded (Ding et al., 2021). The relative luciferase activity was obtained by normalizing the firefly luciferase activity against the internal Renilla luciferase control activity.

### Statistical Analysis

Data were prepared as mean ± SD and statistically analyzed by one-way ANOVA using SPSS version 16.0 software. The Student’s *t*-test was used for comparisons between two experiments. A value of *p* < 0.05 was considered statistically significant.

## Results

### Res Inhibited the Expressions of PRV Early/Late Genes

**Figure 1:**
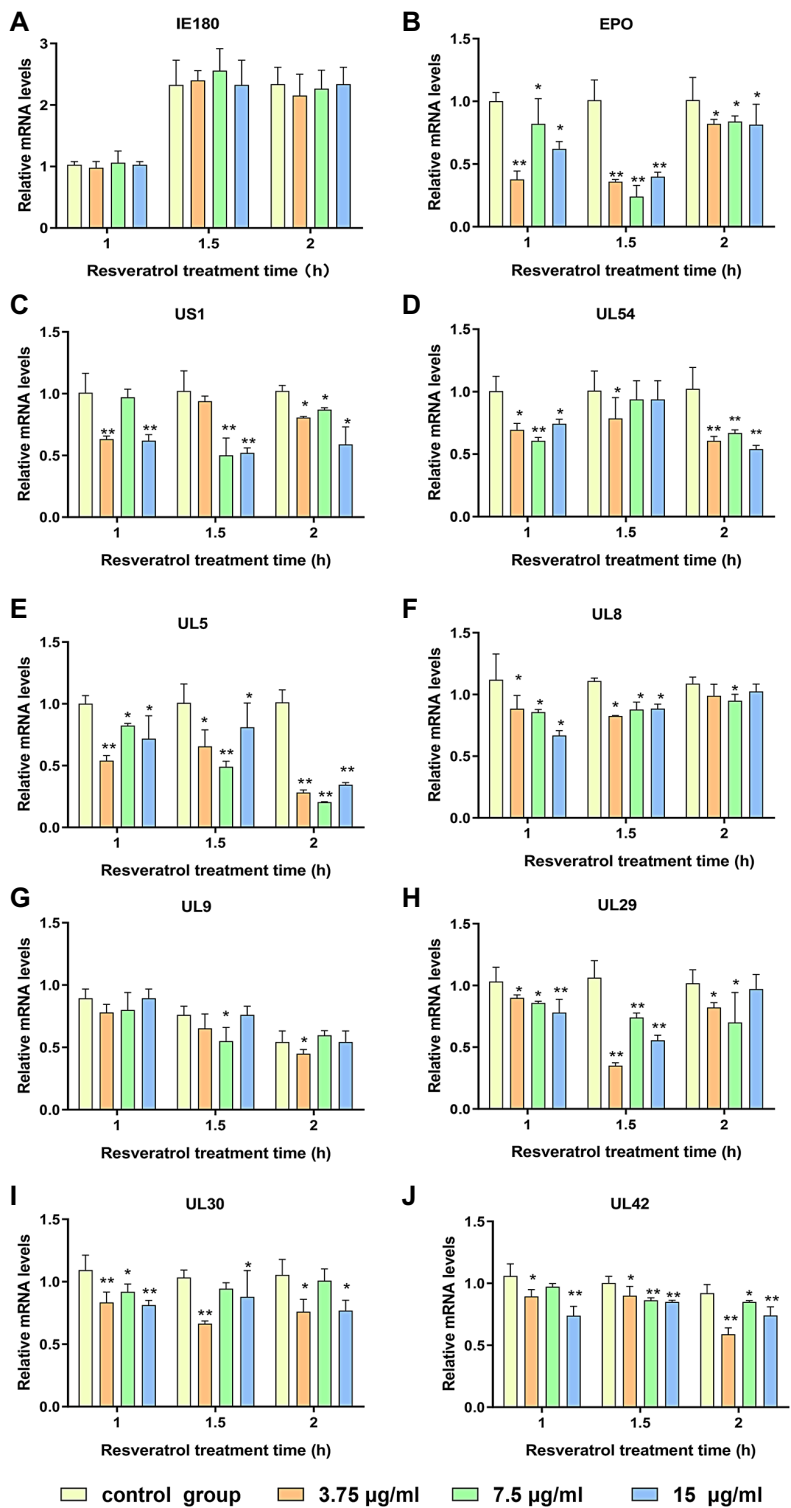
Res inhibit the expressions of pseudorabies virus (PRV) genes. Gene expression levels of PRV were quantified by RT-PCR at 1, 1.5, and 2 h in the presence or absence of Res (3.75, 7.5, and 15 μg/ml, respectively). **(A)** Effect of Res on the mRNA expression level of immediate early gene IE180. **(B–D)** Effects of Res on mRNA expression levels of early genes. **(E–J)** Effect of Res on mRNA expression levels of genes necessary for PRV replication. Values are presented as means ± SD (*n* = 3), ^*^p < 0.05 vs. control group; ^**^p < 0.01 vs. control group.

The PK-15 cells were infected with PRV (MOI = 5). Then, the Res at the concentration of 3.75, 7.5, and 15 μg/ml, respectively, was added. The transcriptional levels of genes, including immediate early gene, early gene, and other genes necessary for PRV replication, were evaluated by RT-PCR. As shown in Figure 1, the immediate early gene expression level (IE180) did not significantly change after Res treatment compared with the control group (Figure 1A). However, the expression levels of early genes (EPO, US1, and UL54) and genes essential for PRV replication (UL5, UL8, UL9, UL29, UL30, and UL42) all showed a decreasing trend in the presence of Res (Figures 1B–J). The results indicated that the Res could not reduce the transcriptional levels of IE180 but significantly inhibit the transcriptional levels of the early and late genes of PRV. It is suggested that Res may affect the function of IE180 protein.

### Res Did Not Affect the Expression of IE180 Protein

**Figure 2:**
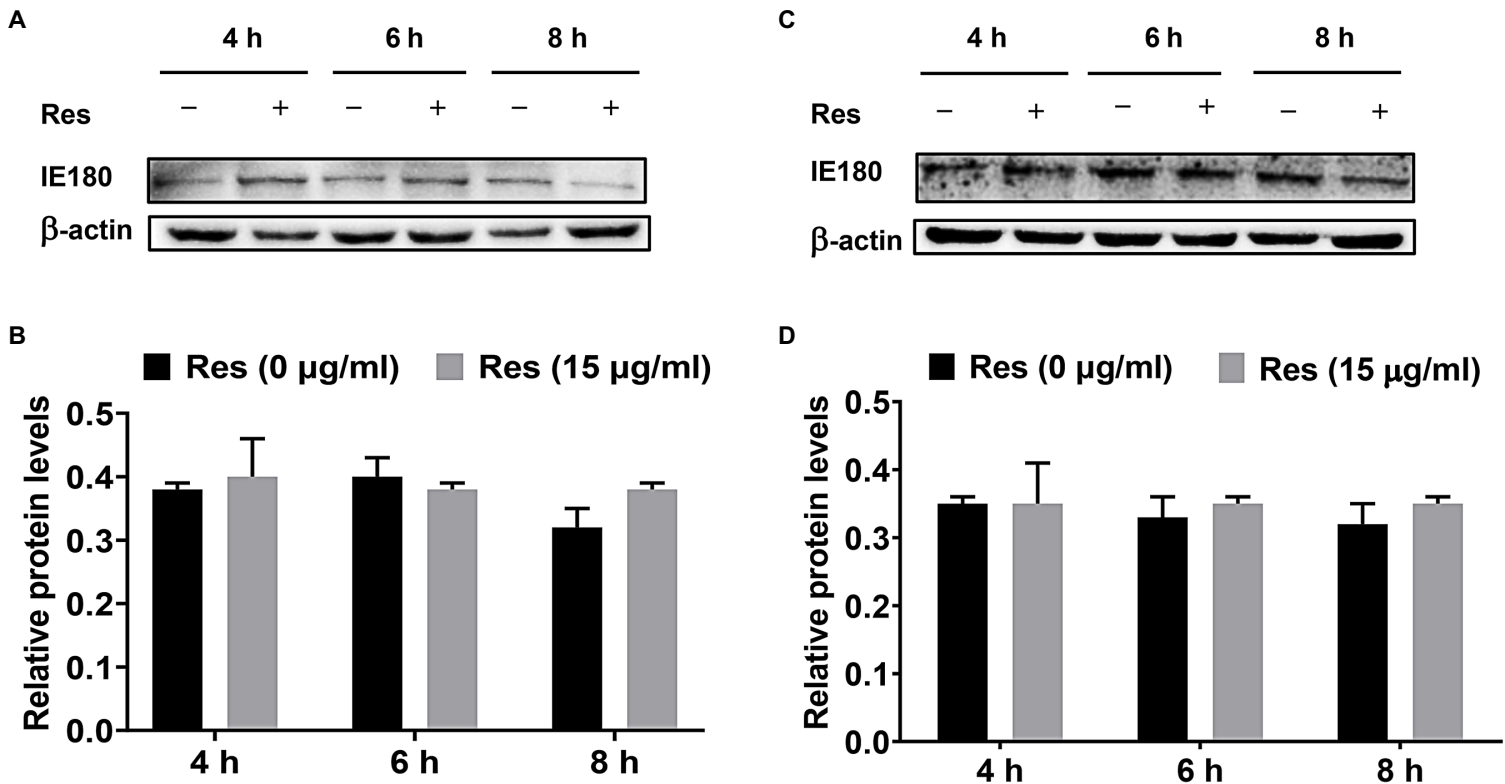
Effect of Res on IE180 protein expression. **(A,B)** PK-15 cells were infected with PRV (MOI = 5) for 1 h, followed by treatment with or without 15 μg/ml of Res at a different time (4, 6, and 8 h). **(C,D)** In the over-expression assay, the recombinant pIE180 was transfected into cells for 24 h followed by infection with PRV (MOI = 5) for 1 h. The cells were then treated with or without Res (15 μg/ml) at various times (4, 6, and 8 h). The expressions of IE180 protein were performed by western blotting and were corrected according to the levels of β-actin. Graphs show mean ± SD (*n* = 3).

The PK-15 cells were infected with PRV (MOI = 5), followed by treatment with Res (15 μg/ml), and the cells were collected to detect the expression level of IE180 protein. Moreover, the recombinant pIE180 was cloned and transfected in PK-15 cells for over-expression. Whether Res could affect the expression level of IE180 protein was studied. The results are exhibited in Figure 2. Compared with the control group, treatment with Res had no significant difference in the expression level of IE180 protein at different time points (*p* > 0.05). These results revealed that Res did not affect the expression of IE180 protein.

### Res Inhibited Nuclear Localization of IE180 Protein

**Figure 3:**
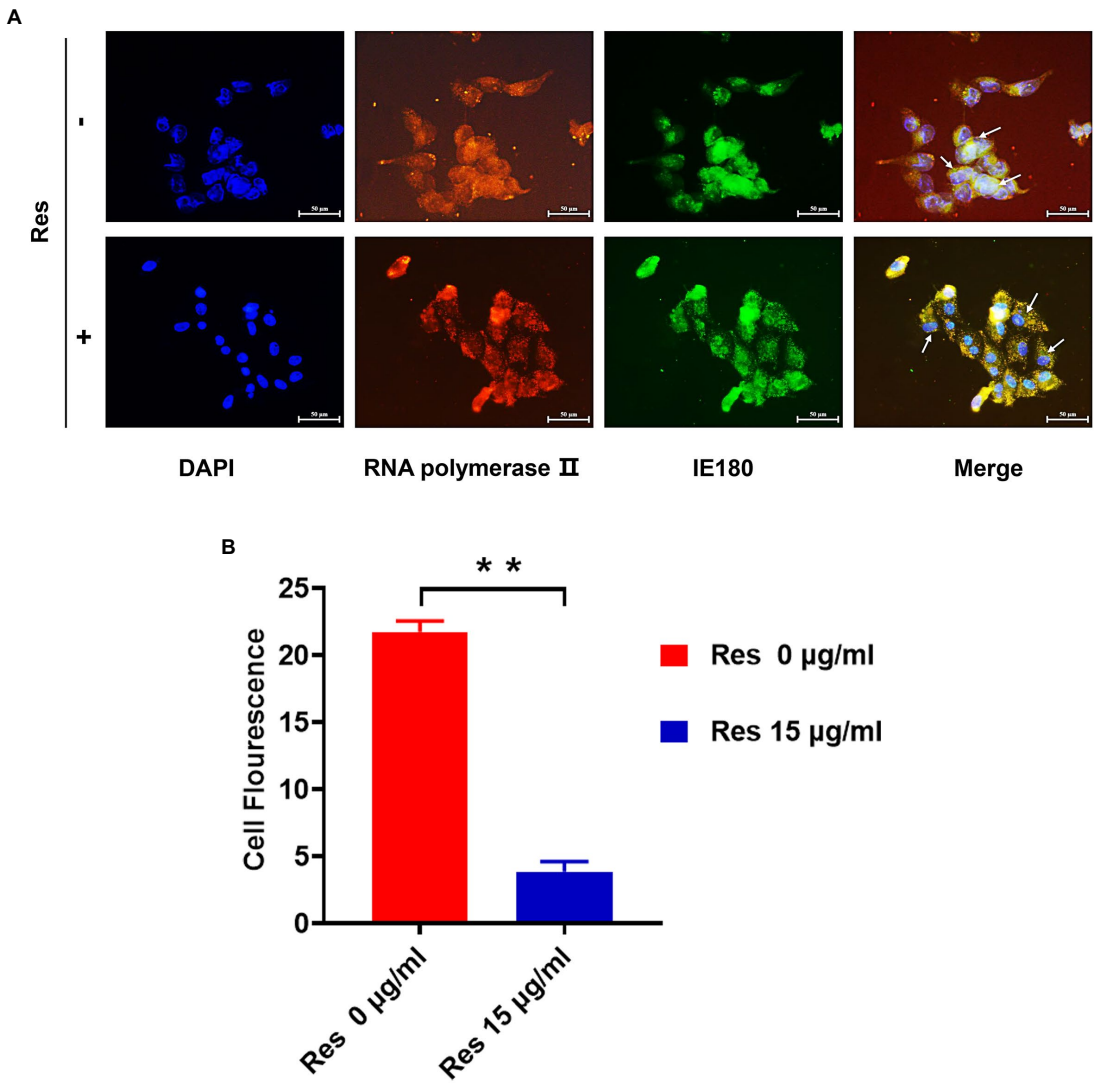
The content of IE180 protein in the nucleus was decreased by Res. **(A)** The PK-15 cells were infected with PRV (MOI = 5) in the absence or presence of Res (15 μg/ml). Immunofluorescence was performed using anti-IE180 (green) and anti-RNA polymerase II (red). Nuclei were stained with DAPI. **(B)** The average fluorescence intensity ratios were calculated, and statistical analysis was performed using the Student’s *t*-test, ^**^p < 0.01. Values are means ± SD (*n* = 3).

It was reported that the IE180 protein is synthesized up until 2.5 post-infection and located in the nucleus (Thapa et al., 2019). IE180 protein expressed in the cytoplasm is translocated to the nucleus and is required for the efficient transcription of viral early-late genes (Zhou et al., 2020). Immunofluorescence assays were performed to assess the effect of Res on IE180 subcellular localization. As shown in Figure 3, in PRV-infected cells, IE180 protein was mainly located in the nucleus. Interestingly, the treatment of resveratrol inhibited the nuclear localization of IE180 protein. These results suggest that Res could affect the transportation of IE180 protein to the nucleus.

### Res Suppressed IE180 Protein Transcriptional Activation Activity

**Figure 4:**
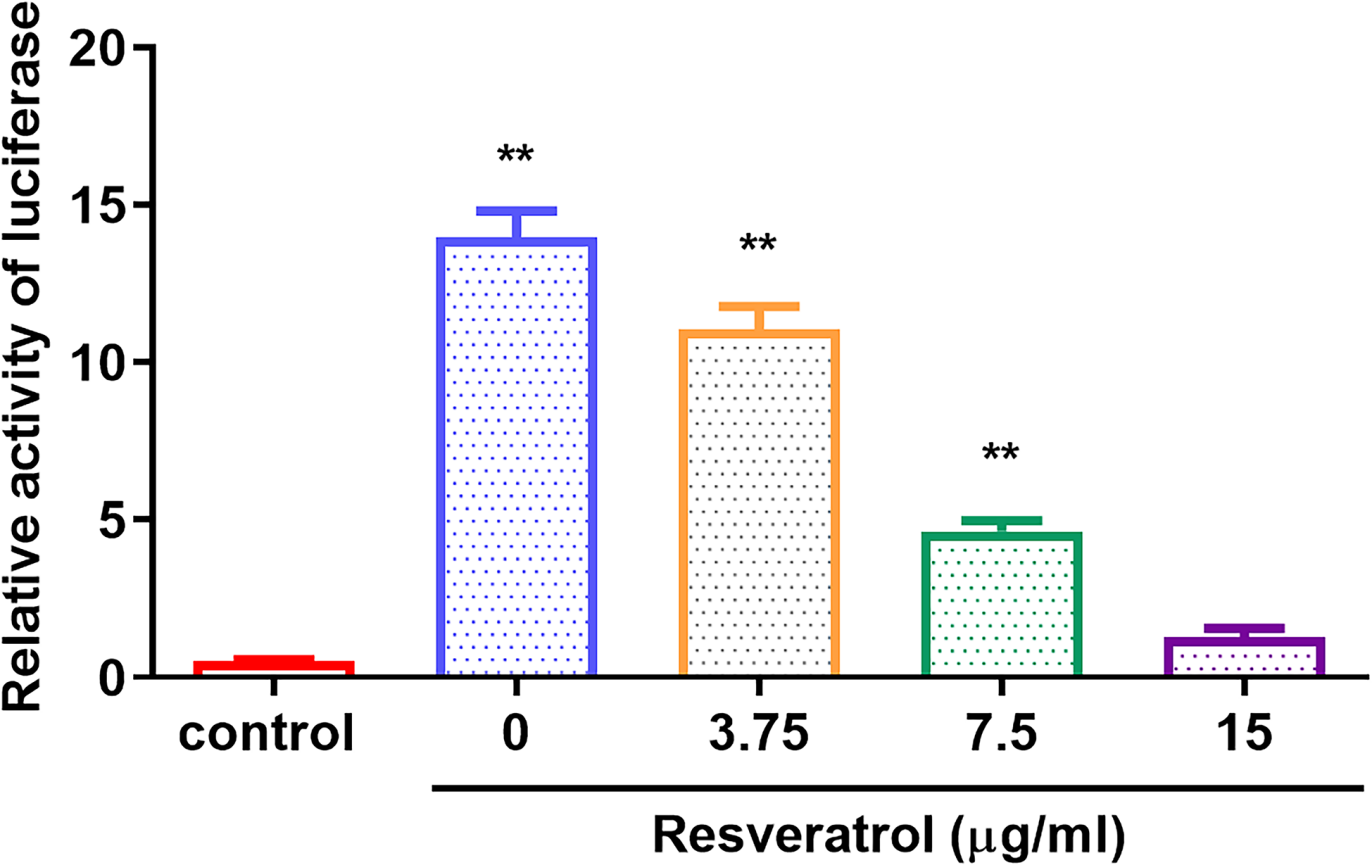
The effects of Res on transcriptional activation of IE180 protein by dual-luciferase assay. The 293 T cells were co-transfected with the effector plasmid pcDNA3.1(+) or pIE180, the reporter plasmid pGL3-TK, and the internal control plasmid pRL-TK. After incubation for 24 h, the Res was added to the plate to treat for 48 h, and relative luciferase activity was measured (*n* = 3). ^**^p < 0.01 vs. control group.

**Figure 5:**
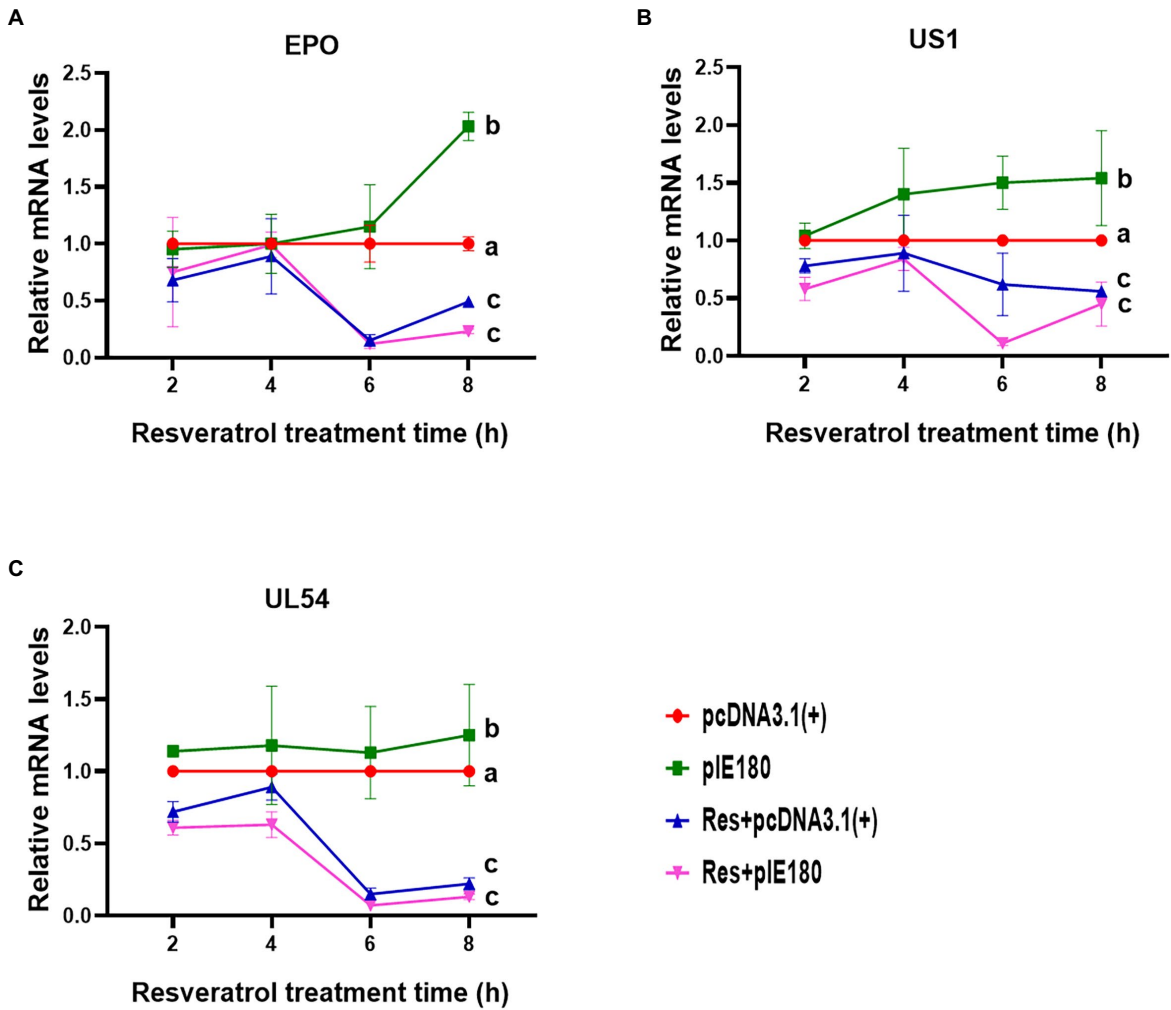
Effects of Res on early gene expressions after transfection with pIE180. **(A)** Effects of Res on early gene (EPO) expressions after transfection with pIE180. **(B)** Effects of Res on early gene (US1) expressions after transfection with pIE180. **(C)** Effects of Res on early gene (UL54) expressions after transfection with pIE180.

The replication initiation of PRV depends on the transcriptional activation of IE180. As shown in Figure 4, compared with the control group, the relative activity of luciferase was reduced by Res in a dose-dependent manner with a range from 0 to 15 μg/ml (*p* < 0.01). The results suggested that Res inhibited the transcriptional activation of IE180 protein. In addition, whether the expressions of early genes under over-expression of IE180 were affected by Res was also examined. As shown in Figure 5, over-expression of IE180 significantly increased the expressions of early genes (EPO, US1, and UL54). After Res-treatment, the expressions of these genes were significantly down-regulated. These results suggested that Res could inhibit PRV replication by inhibiting the transcriptional activation function of IE180 protein.

### The Binding Sites of Res With IE180 Protein Were Analyzed by Molecular Docking

**Figure 6:**
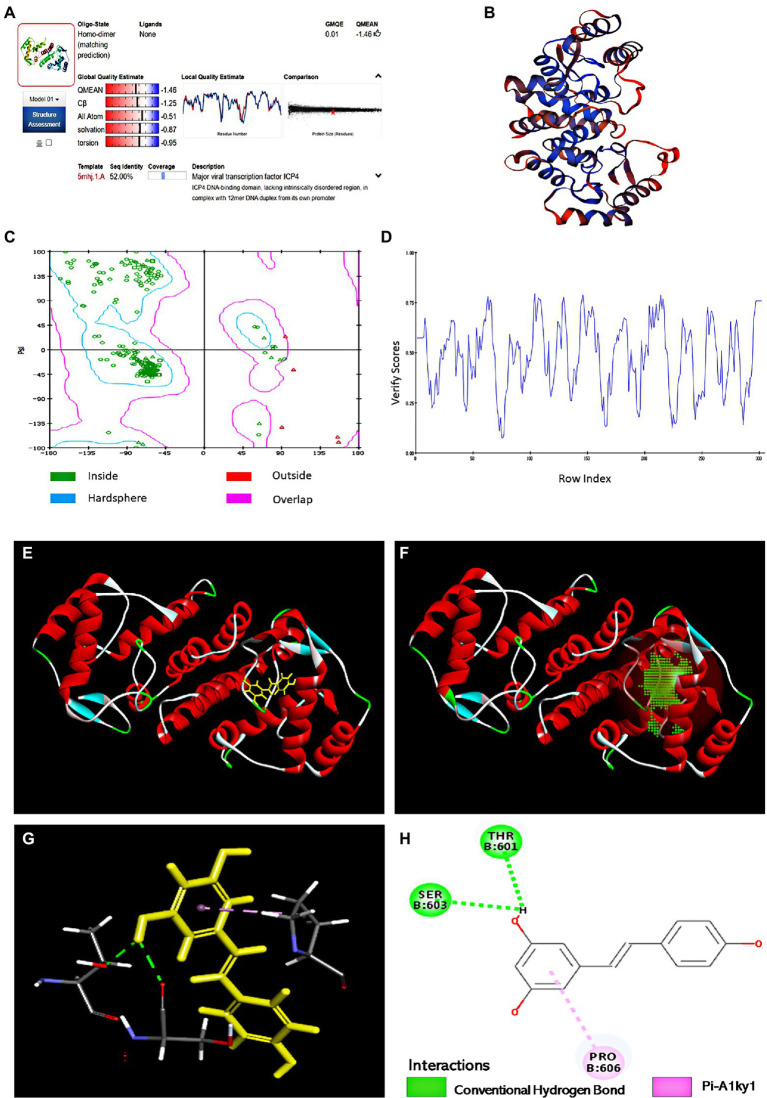
The binding sites of Res with IE180 by molecular docking. **(A)** Alignment of the amino acid sequence of IE180 and ICP4 (5mhj.1. A). **(B)** The 3D structure of IE180 is conducted by homologous. **(C)** Ramachandran plots of the homologous mode of IE180 (Blue: the best region; Purple: the appropriate region; Red: the barely permitted region; and White: the disallowed region). **(D)** Profile 3D plot IE180 3D structure. **(E)** The 3D structure of IE180 includes an active docking site. **(F)** 3D docking mode between Res and IE180simulated by Discovery Studio. **(G)** The interaction of IE180 with Res; the green and purple colors represent H-bond and Pi-Alkyl, respectively. **(H)** 2D schematic interaction diagram between IE180 and Res; the green color and purple color represent H-bond and Pi-Alkyl, respectively.

Hit molecules that showed the expected interactions with the critical amino acids present in the protein’s active site may show potent antagonist properties towards IE180 protein (Mpiana et al., 2020). In our study, Molecular docking was performed to predict and visualize the interaction between Res and IE180. However, the crystallographic structure for IE180 protein has not been solved yet. Therefore, we used homology modeling to construct the three-dimensional structure of IE180 based on its known amino acid sequence and the known structures of a closely related protein. The online Swiss-MODLE protein tertiary structure homology modeling database was used to predict the 3D structure of IE180, which indicates that the sequences identity with homologous proteins ICP4 (PDB ID: 5mhj.1. A; Figure 6A) was 52%.

Regarding the minimum sequence similarity limit in homology modeling, there are ambiguities about the exact value, but >25% suggests that the template and target will take a similar 3D structure (Muhammed and Aki-Yalcin, 2019). The 3D structure of IE180 built using the Modeling program is shown in Figure 6B. The structure was evaluated using *X-ray* structure, *Ramachandran Plot*, and *Profile-3D* model modules of Discovery Studio software. The results of the constructed X-ray structure indicated that the percentage of amino acid residues in the allowed, marginal, and disallowed regions were 97.6%, 2.4%, and 0.00%, respectively. It showed that the configuration has high reliability and can be further used for molecular docking. The Ramachandran Plot (Ramachandran Plot) is a visualization method used to describe whether the dihedral Angle phi (φ) and psi (ψ) of amino acid residues in protein structure are in a reasonable region. It can also reflect whether the conformation of the protein is reasonable (Dushanan et al., 2020). Most of the amino acid residues in the IE180 had energetically favorable torsion angles (Figure 6C; the blue area in Ramachandran Plot is the optimal area), which revealed that the constructed protein structure is reasonable. Profiles-3D is a model evaluation program, and the higher the validation score of amino acid residues, the greater the reliability of the homologous model (Dong et al., 2018). All amino acid residues did indeed have positive verify scores (Figure 6D). In a word, all results indicated that the homologous modeling structure constructed was reasonable and therefore suitable for carrying out the molecular docking study.

The potential binding sites based on protein cavities are shown in Figure 6E using molecular docking protocol. Binding energy (Kcal/mol) is often regarded as the binding affinity between ligands and their corresponding target molecules. Lower binding energy indicates a higher affinity of ligands with their respective target molecule (Rahman et al., 2021). In this study, the arrangement that gave the lowest energy score (CDOCKER energy −24.85 kcal/mol; CDOCKER interaction energy −33.62 kcal/mol) was taken to represent the binding interaction Res with IE180. Combined 3D docking mode (Figures 6F,G) and the 2D schematic diagram (Figure 6H), It clearly showed that Res was inserted into the active site of the IE180 and interacted with three amino acids (Thr601, Ser603, and Pro606), suggesting that these are possible interaction sites. Res showed hydrogen bonding with Thr601 and Ser603 with bond lengths of 2.17 and 2.27 Å, respectively. One Pi-Alkyl was found between Res and specific amino acid residues (Pro606 4.24 Å), which helped stabilize the resveratrol bounded with the active residues of the subunit. Altogether, the results revealed that Res docked well with IE180 and the potential binding site were Thr601, Ser603, and Pro606.

### Res Was Insensitive to Binding Site Mutants

**Figure 7:**
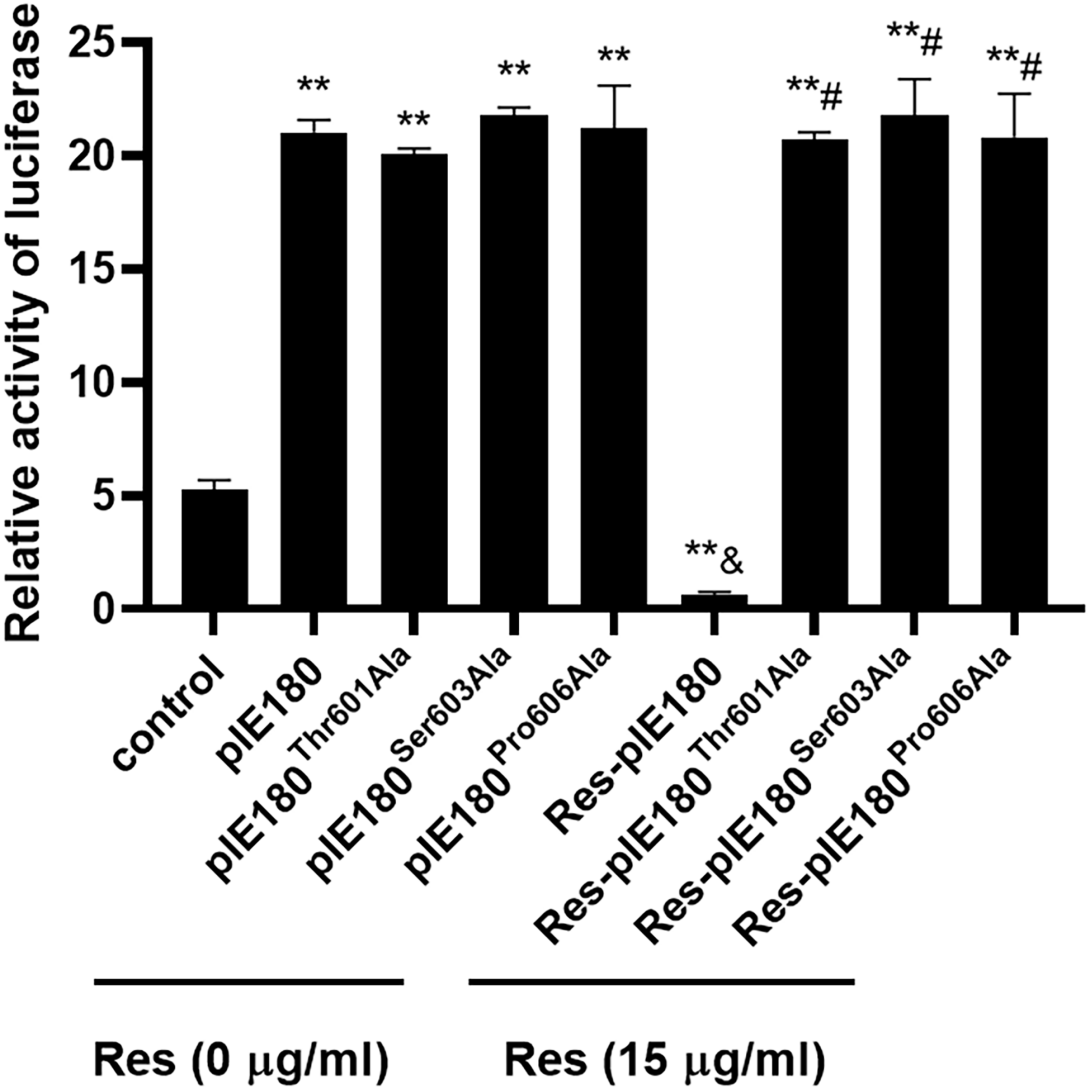
The effects of Res on IE180 activation were detected using the dual-luciferase assay. The 293 T cells were co-transfected with the effector plasmid pcDNA3.1(+)/pIE180//pIE180^Thr601Ala^/pIE180^Ser603Ala^/pIE180^Pro606Ala^, the reporter plasmid pGL3-TK, the internal control plasmid pRL-TK, respectively. After incubation for 24 h, the Res (15 μg/ml) was added to the plate to treat for 48 h, and relative luciferase activity was measured. ^**^p < 0.01 represents a significant difference compared to the control group; ^#^*p* < 0.01 represents a significant difference compared to the Res-pIE180 group; and ^&^*p* < 0.01 represents a significant difference compared to the pIE180 group (*n* = 3, in each group).

**Figure 8:**
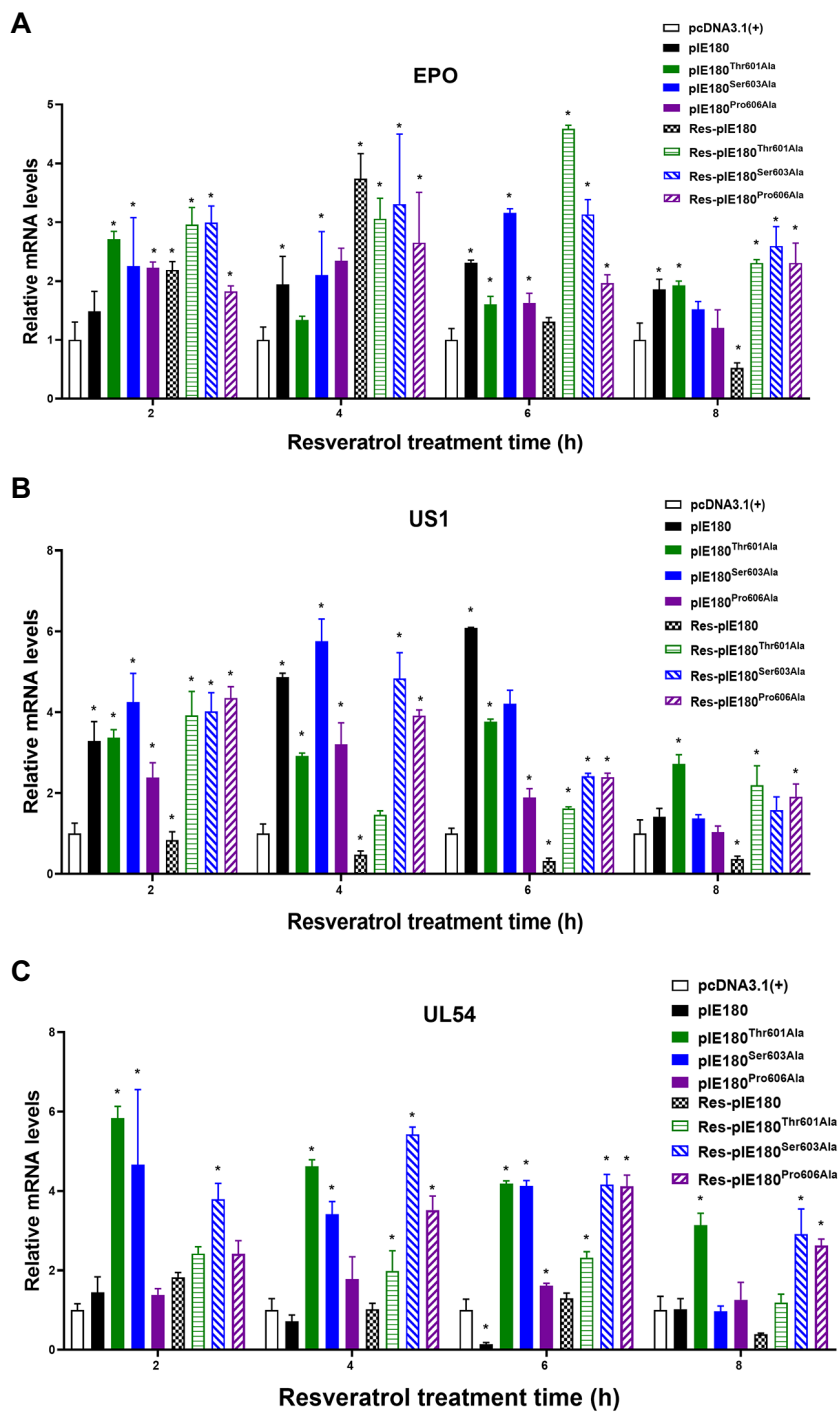
Effect of Res on early gene expression after transfection with pcDNA3.1(+)/pIE180/pIE180^Thr601Ala^/pIE180^Ser603Ala^/pIE180^Pro606Ala^. **(A)** Effect of Res on early gene (EPO) expression. **(B)** Effect of Res on early gene (US1) expression. **(C)** Effect of Res on early gene (UL54) expression. ^*^p < 0.05 represents a significant difference compared to the pcDNA3.1(+) group (*n* = 3).

The binding sites obtained by molecular docking were further confirmed by site-directed mutagenesis. The pcDNA3.1 (+)/pIE180/pIE180^Thr601Ala^/pIE180^Ser603Ala^/pIE180^Pro606Ala^ with pGL-TK were co-transfected, respectively, to detect their luciferase activity. The results indicated that luciferase activity was significantly increased in the pIE180 group and three mutant groups (*p* < 0.01) compared with the control group. However, there was no significant difference between the wild-type pIE180 group and the three mutation groups (*p* > 0.05). After Res treatment, compared with the control group, the luciferase activity in the wild-type pIE180 group was significantly decreased, while the luciferase activity in each mutant group was significantly increased (*p* < 0.01; Figure 7). In addition, luciferase activity was significantly decreased in Res-pIE180 group compared with pIE180 group (*p* < 0.01). However, there were no significant differences among mutant groups with Res treatment when compared with those without Res treatment (*p* > 0.05; Figure 7). The results showed that the three mutants were insensitive to Res. To further verify whether Res could affect early gene transcription levels in the presence of these mutants, the mRNA expression levels of early genes (EPO, US1, and UL54) were detected. As shown in Figure 8, compared with the control group, the levels of the early genes in the wild-type group and the mutant groups were significantly increased without Res treatment. In the presence of Res, mRNA levels of the early genes in the wild-type pIE180 group were significantly lower than those in the untreated control group, while the levels in each mutant group were higher than those in the wild-type pIE180 group. Altogether, these data suggested that Res may bind to the residues of IE180, Thr601, Ser603, and Pro606.

## Discussion

Resveratrol is a natural component of certain foods, such as grapes and red wine. Many studies have demonstrated that resveratrol has antiviral effects, such as herpes viruses, enteroviruses, and HIV (Annunziata et al., 2020). Our previous studies showed that resveratrol inhibits PRV infection *in vivo* and *in vitro*.

The IE180, the only IE gene of PRV, is a transcription factor and is considered to be essential for the transcription of all known genes (Tombácz et al., 2017). IE180 is highly similar to the IE proteins of other *Alphaherpesviruses* such as ICP4 of HSV-1, IE140 of VZV, IE1 of equine herpesvirus1, and p180 of bovine herpesvirus1 (Csabai et al., 2017). All herpesviruses have the same regulatory mechanism. Thus, IE180 protein as an anti-PRV target in the present study is also helpful for antiviral drug development against other *Alphaherpesviruses*. The transcription of the IE180 gene did not require any viral protein synthesis and was synthesized 40 min after infection (Pomeranz et al., 2005; Guo et al., 2009; Zhang et al., 2020). The early/late genes’ transcription depends on IE180 protein, and the early/late proteins are necessary for viral DNA replication (Tombácz et al., 2012). Our findings demonstrated that Res inhibited early/late gene transcription while did not inhibit IE180 mRNA expression. IE180 is a phosphorylated protein localized in the nucleus of an infection. It is translated in the cytoplasm and then returns to the nucleus to interact with DNA to activate transcription (Sparks-Thissen and Enquist, 1999). According to the characteristic of IE180, we speculated that resveratrol might inhibit the function of IE180 protein, thus blocking the downstream gene expression.

Furthermore, we further evaluated whether Res could affect the content of IE180 protein. In PRV-infected cells, the expression of IE180 protein was not affected by Res. Then, IE180 was transfected, and Res did not affect the over-expression of IE180 protein. Intriguingly, the results from immunofluorescence assays revealed that Res suppressed IE180 protein entering into the nucleus. As a regulatory protein, IE180 needs to return to the nucleus to play the role of transcriptional activation, which is a very critical step for downstream gene transcription (Taharaguchi et al., 1995). These data indicate that Res has no effects on the mRNA transcription and protein synthesis of IE180, and it may inhibit the transportation of IE180 protein to the nucleus.

After herpesvirus infection, viral gene expression occurs in a coordinated and regulated sequence. Immediately after infection, a set of viral gene products requiring no prior viral protein synthesis is named immediately early gene products, such as IE180, ICP4, and ICP27. These proteins are largely responsible for activating early gene transcription and remolding the microenvironment to support viral replication (Han et al., 2020). IE180 has been identified as a potential transcriptional activator. Like most typical activators, IE180 contains a separate DNA-binding domain and a trans-activation domain (Wu and Wilcox, 1991). Therefore, whether Res also affected the transcriptional activation of IE180 was tested. The present study revealed that in the presence of resveratrol, the transcriptional activation of early gene promoters was reduced by IE180. This phenomenon was also seen in the mRNA expression levels of the early genes. These results proved that IE180 played an indispensable role in the target of Res-treatment. Based on the above results, we speculated that Res might bind to IE180 protein, leading to the blocked transcriptional activation function of IE180, thus affecting the transcription of downstream genes and ultimately inhibiting the replication process of the virus.

It is unknown whether resveratrol binds to IE180 protein and the interaction sites. Molecular docking was a powerful tool to visualize the interactions between receptor and ligand molecules (Joshi et al., 2021). Therefore, molecular docking technology was performed to analyze the possible binding sites. The results showed that the Res formed a hydrogen bond with the residuesThr601/Ser603 (N-H-Thr601and Ser603) and formed a Pi-A1ky1 bond with Pro606. Meanwhile, the high interaction energy also showed that resveratrol bonds tightly to IE180. IE180 is a very complex protein in both structure and function. For the transcriptional regulation of IE180, only the first 1,081 amino acids are involved in transcriptional activation and self-regulation (Nie et al., 2020). Remarkable, the three amino acid binding sites of resveratrol and IE180 were all located in 1–1,081 amino acids. These results further supported the previous speculation that resveratrol may bind to IE180 to block its transcriptional activation function.

To further prove this inference, we continued to measure whether resveratrol affected its transcriptional activation after the point mutation of the three amino acids (Thr601, Ser603, and Pro606). The results demonstrated that the three mutants showed strong transcriptional activation of early gene promoters regardless of Res’s presence, suggesting that Res did not interact with IE180 due to a mutation in the binding site. These results suggested that Thr601, Ser603, and Pro606 were the binding sites of Res with IE180.

**Figure 9:**
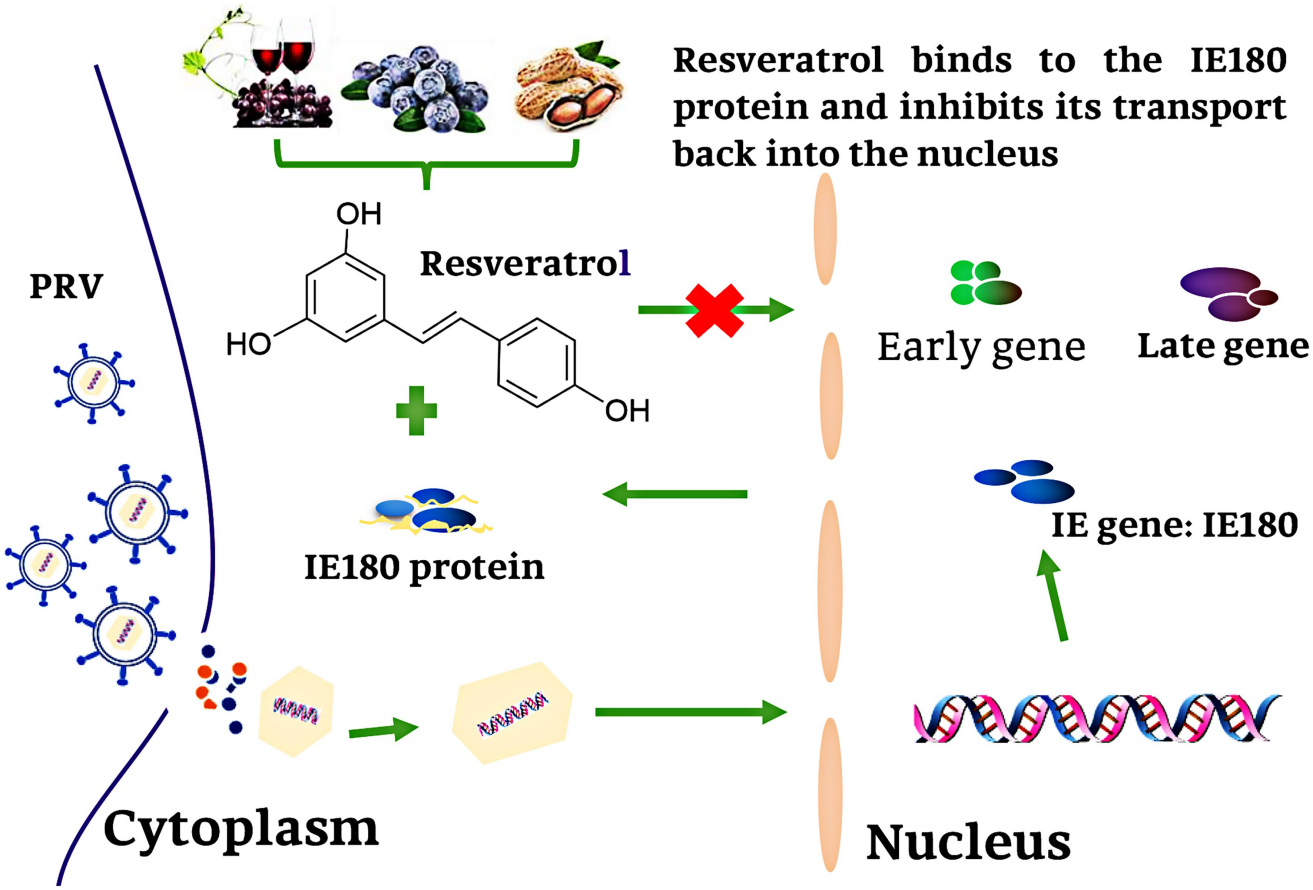
Schematic presentation of possible the Res anti-PRV molecular mechanism. Res binds to the amino acid of IE180 protein (Thr601, Ser603, and Pro606), thus affecting its transcriptional activation function, leading to the blocked downstream gene expression.

In summary, our results demonstrated that Res did not affect the mRNA and protein expression levels of IE180. However, the nuclear localization and transcriptional activation ability of IE180 protein were inhibited by Res. Res binds to the amino acids of IE180 protein (Thr601, Ser603, and Pro606), thus affecting its transcriptional activation function, leading to the blocked downstream gene expression. The antiviral mechanism of Res against PRV is summarized in Figure 9. This study represents a new insight into antiviral drug development against herpes viruses.

## Data Availability Statement

The original contributions presented in the study are included in the article/Supplementary Material; further inquiries can be directed to the corresponding authors.

## Author Contributions

ZY and RJ: conceptualization. XC, XS, and LL: methodology and writing-original draft preparation. YC, YZ, and HW: formal analysis. ZY, RJ, and XZ: writing, reviewing, and editing. ZY: funding acquisition. LZ, HT, and CL: project administration. All authors contributed to the article and approved the submitted version.

## Funding

This research was financially supported by the Program Sichuan Veterinary Medicine and Drug Innovation Group of China Agricultural Research System (SCCXTD-2020-18) and the Science and Technology Project of Sichuan Province (2021NZZJ0021).

## Conflict of Interest

The authors declare that the research was conducted in the absence of any commercial or financial relationships that could be construed as a potential conflict of interest.

## Publisher’s Note

All claims expressed in this article are solely those of the authors and do not necessarily represent those of their affiliated organizations, or those of the publisher, the editors and the reviewers. Any product that may be evaluated in this article, or claim that may be made by its manufacturer, is not guaranteed or endorsed by the publisher.
