# Supplementary material for: Resveratrol Inhibits Pseudorabies Virus Replication by Targeting IE180 Protein
Source: Front Microbiol. 2022 Jun 2;13:891978. doi: 10.3389/fmicb.2022.891978 (PMC9203040; doi:10.3389/fmicb.2022.891978)
Supplement: Supplementary file 1 [file Data_Sheet_1.PDF]

Supplementary Material

Resveratrol inhibits pseudorabies virus replication by targeting IE180 protein

Xiangxiu Chen [a†], Xu Song [a†], Lixia Li [a†], Yaqin Chen [a], Renyong Jia [b*], Yuanfeng Zou [a], Hongping Wan [a], Ling Zhao [a], Huaqiao Tang [a], Cheng Lv [a], Xinghong Zhao [a] and Zhongqiong Yin [a*]

[†] These authors have contributed equally to this work and share the first authorship

[a] Natural Medicine Research Center, College of Veterinary Medicine, Sichuan Agricultural University, Chengdu 611130, China

[b] Key Laboratory of Animal Disease and Human Health of Sichuan Province, Sichuan Agricultural University, Chengdu 611130, China

[*] Corresponding author: Renyong Jia, Zhongqiong Yin

Tel: +86 028 86291176; Fax: +86 835 2885302

E-mail address: jiary@sicau.edu.cn；yinzhongq@163.com

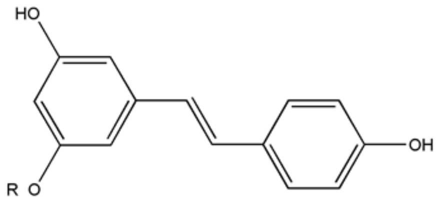

R=H Trans(E) Resveratrol

R=glucosides Cis(Z) Resveratrol

Supplementary Figure S1| Chemical structure of resveratrol.

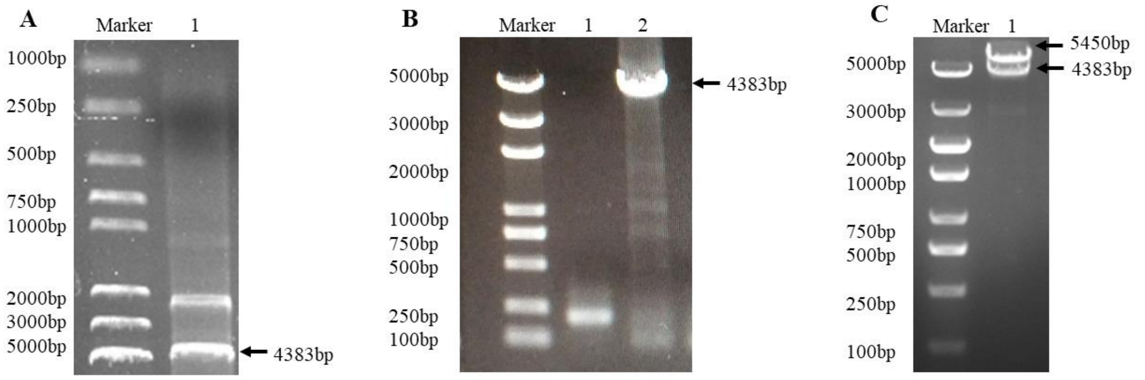

Supplementary Figure S2| Construction of recombinant plasmid pIE180.
(A) PCR amplification of IE180 gene. (B) Colony identification of IE180 gene.
(C) Identification of recombinant plasmid pIE180 by double enzyme digestion.

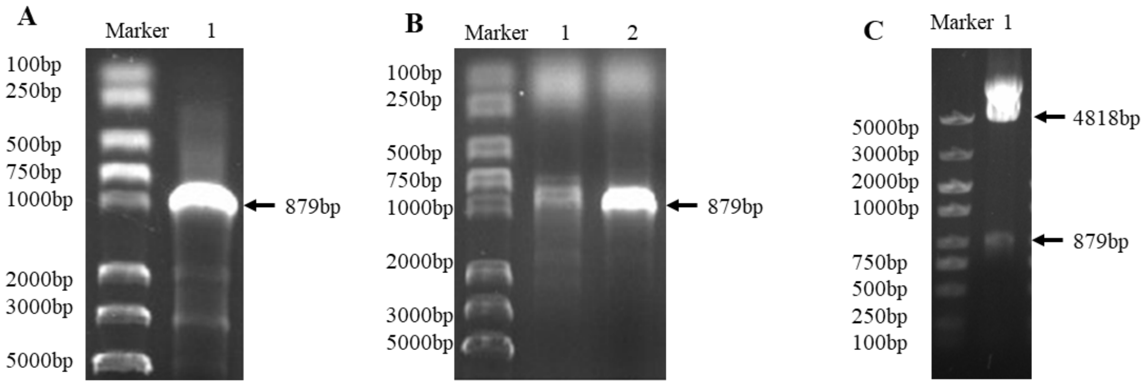

Supplementary Figure S3| Construction of recombinant plasmid pGL3-TK.
(A) PCR amplification of TK gene. (B) Colony identification of TK gene.
(C) Identification of recombinant plasmid pGL3-TK by double enzyme digestion.

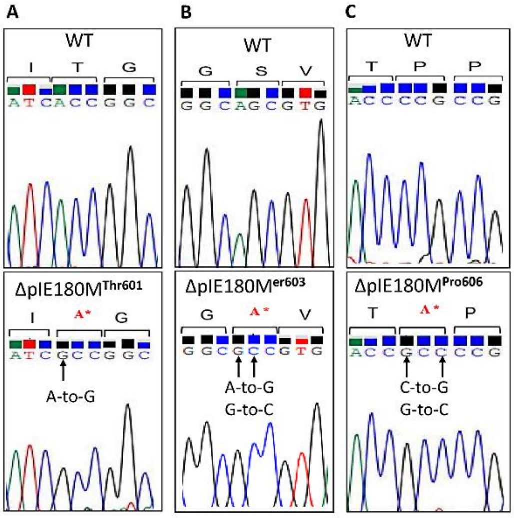

Supplementary Figure S4| Nucleotide and amino acid sequences are shown below the chromatograms.
（A） The mutant allele shows a A-to-G transversion resulting in a Thr to Ala missense mutation at amino acid position 601. (B) The mutant allele has two A-to-G， G-to-C transition resulting in a Ser to Ala missense mutation at amino acid position 603. (C) The mutant allele has two C-to-G， G-to-C transition resulting in a Pro to Ala missense mutation at amino acid position 606.

Supplementary Table S1| Primers for plasmid construction

Specificity	Sequence (5'-3')
IE180	F: CCAGATTACGCTCTTAAGCTTATGGCCGACGATCTCTTTGA
	R: CCACACTGGACTAGTGGATCCTCAGCGGAGCAGCAGGTAG
TK	F: CGTACGGCTAGCATCATCTGATTGGCTCGCTA
	R: CGCTAGAAGCTTGGTCTCGATCGGTTCTCG

Supplementary Table S2| List of all plasmids

plasmid	source
pcDNA3.1 (+)	Miaoling, Wuhan China
pIE180	Construction of plasmid
pGL3-Basic-Vector	Promega, USA
pRL-TK	Promega, USA
pGL3-TK	Construction of plasmid
$pIE180^{Thr601Ala}$	Construction of plasmid
$pIE180^{Ser603Ala}$	Construction of plasmid
$pIE180^{Pro606Ala}$	Construction of plasmid

Supplementary Table S3| Oligos for primers

Primers for real-time PCR

Specificity	Sequence (5'-3')	
IE180	F: CATCGTGCTGGACACCATCGAG	R: ACGTAGACGTGGTAGTCCCCCA
EPO	F: GGGTGTGAACTATATCGACACGTC	R: TCAGAGTCAGAGTGTGCCTCG
US1	F: AGCTCAACGAGCGCGACGTCTA	R: CGGAAGCTAAACTCGGACGCGA
UL54	F: TGCAGCTACACCCTCGTCC	R: TCAAAACAGGTGGTTGCAGTAAA
UL5	F: CCTTCATGAGCATCTTGCCG	R: ACCGCGCGATGGTCAT
UL8	F: CCGCTGATCCTGCCCTG	R: GAAGATGGGCTCCATGTGG
UL9	F: CAAGTTCAAGCACCTGTTCGA	R: TGAGGCTGTCGTTGACGC
UL29	F: CTGATCCTGCGCTACTGCG	R: ACTGCATCGTGATCCCCG
UL30	F: TCATCACGAAGAAGAAGTACATCGG	R: CCTTCATGAGCATCTTGCCG
UL42	F: GCTCCCCGAGCGTCG	R: CATGATGCAGTAGTCGTTGAACTC
UL52	F: CGCGCAACTTTCACTTCCACGCA	R: TGCGCTCGAAGAAGCTCTCGTA
β-actin	F: GGACTTCGAGCAGGAGATGG	R: AGGAAGGAGGGCTGGAAGAG

Supplementary Table S4| Primer sequence for the mutants

mutant	Sequence (5'-3')
pIE180^{Thr601Ala}	F: TCCTTCATCGCCGGCAGCGTGACCCCGCCGCT
	R: TCACGCTGCCGGCGATGAAGGAGCCGTGGCCGT
pIE180^{Ser603Ala}	F: ATCACCGGCGCCGTGACCCCGCCGCTGCCGCAC
	R: TCACGCTGCCGGCGATGAAGGAGCCGTGGCCGT
pIE180^{Pro606Ala}	F: AGCGTGACCGCCCCGCTGCCGCACATCGGGGA
	R: GGCAGCGGGGCGGTCACGCTGCCGGTGATGAA
