# Supplementary material for: Resveratrol Inhibits Pseudorabies Virus Replication by Targeting IE180 Protein
Source: Front Microbiol. 2022 Jun 2;13:891978. doi: 10.3389/fmicb.2022.891978 (PMC9203040; doi:10.3389/fmicb.2022.891978)
Supplement: Supplementary file 2 [file Data_Sheet_2.ZIP › Raw Data/Figure 1 (data sheet).pdf]

IE180 gene relative mRNA level

group	Res (0 µg/ml)	Res (3.75 µg/ml)	Res (7.5 µg/ml)	Res (15 µg/ml)
**1 h**	1.01	1.07	1.27	1.08
**1 h**	1.09	0.87	0.89	0.98
**1 h**	0.99	0.99	1.03	1.03
**1.5 h**	2.19	2.6	2.81	2.77
**1.5 h**	2.02	2.38	2.15	2.21
**1.5 h**	2.78	2.29	2.72	2
**2 h**	2.65	2.54	2.6	2.65
**2 h**	2.12	2.03	2.2	2.25
**2 h**	2.25	1.88	2.01	2.12

EPO gene relative mRNA level

group	Res (0 µg/ml)	Res (3.75 µg/ml)	Res (7.5 µg/ml)	Res (15 µg/ml)
**1 h**	0.92	0.34	0.83	0.65
**1 h**	1.04	0.47	0.83	0.65
**1 h**	1.03	0.34	0.8	0.55
**1.5 h**	0.9	0.34	0.2	0.43
**1.5 h**	0.94	0.38	0.18	0.39
**1.5 h**	1.19	0.37	0.35	0.38
**2 h**	1.22	0.85	0.88	1
**2 h**	0.87	0.8	0.8	0.72
**2 h**	0.95	0.81	0.84	0.71

US1 gene relative mRNA level

group	Res (0 µg/ml)	Res (3.75 µg/ml)	Res (7.5 µg/ml)	Res (15 µg/ml)
**1 h**	1.18	0.65	0.99	0.68
**1 h**	0.87	0.63	1.03	0.6
**1 h**	0.98	0.61	0.9	0.58
**1.5 h**	1.02	0.92	0.34	0.56
**1.5 h**	1.18	0.99	0.59	0.51
**1.5 h**	0.87	0.91	0.58	0.49
**2 h**	1.06	0.8	0.89	0.75
**2 h**	0.96	0.81	0.86	0.48
**2 h**	1.04	0.82	0.87	0.55

UL54 gene relative mRNA level

group	Res (0 µg/ml)	Res (3.75 µg/ml)	Res (7.5 µg/ml)	Res (15 µg/ml)
**1 h**	0.86	0.73	0.65	0.78
**1 h**	1.07	0.63	0.59	0.75
**1 h**	1.06	0.7	0.6	0.7
**1.5 h**	1.03	0.99	1.1	1.09
**1.5 h**	0.85	0.7	0.8	0.8
**1.5 h**	1.16	0.68	0.93	0.93
**2 h**	0.85	0.66	0.69	0.56
**2 h**	1.02	0.59	0.68	0.56

**2 h**	1.18	0.58	0.64	0.51

UL5 gene relative mRNA level

group	Res (0 µg/ml)	Res (3.75 µg/ml)	Res (7.5 µg/ml)	Res (15 µg/ml)
**1 h**	1.06	0.58	0.82	0.93
**1 h**	0.93	0.55	0.8	0.67
**1 h**	1.02	0.5	0.83	0.56
**1.5 h**	1.18	0.59	0.52	1.01
**1.5 h**	0.93	0.81	0.44	0.82
**1.5 h**	0.91	0.59	0.52	0.61
**2 h**	1.11	0.28	0.2	0.36
**2 h**	0.91	0.26	0.2	0.36
**2 h**	1.01	0.3	0.2	0.32

UL8 gene relative mRNA level

group	Res (0 µg/ml)	Res (3.75 µg/ml)	Res (7.5 µg/ml)	Res (15 µg/ml)
**1 h**	1.31	0.99	0.88	0.71
**1 h**	0.89	0.86	0.85	0.66
**1 h**	1.16	0.8	0.85	0.64
**1.5 h**	1.13	0.83	0.94	0.94
**1.5 h**	1.09	0.83	0.88	0.87
**1.5 h**	1.13	0.83	0.82	0.86
**2 h**	1.14	1.07	0.98	1.07
**2 h**	1.09	1.02	0.98	0.96
**2 h**	1.04	0.89	0.9	1.02

UL9 gene relative mRNA level

group	Res (0 µg/ml)	Res (3.75 µg/ml)	Res (7.5 µg/ml)	Res (15 µg/ml)
**1 h**	0.97	0.85	0.95	0.91
**1 h**	0.86	0.76	0.77	0.81
**1 h**	0.84	0.73	0.68	0.95
**1.5 h**	0.78	0.77	0.68	0.83
**1.5 h**	0.69	0.54	0.52	0.76
**1.5 h**	0.82	0.64	0.46	0.69
**2 h**	0.6	0.49	0.65	0.45
**2 h**	0.44	0.43	0.59	0.56
**2 h**	0.57	0.44	0.57	0.62

UL29 gene relative mRNA level

group	Res (0 µg/ml)	Res (3.75 µg/ml)	Res (7.5 µg/ml)	Res (15 µg/ml)
**1 h**	1.07	0.91	0.87	0.69
**1 h**	0.9	0.92	0.86	0.74
**1 h**	1.13	0.88	0.86	0.9
**1.5 h**	1.11	0.37	0.74	0.6
**1.5 h**	0.9	0.34	0.78	0.57

**1.5 h**	1.17	0.33	0.7	0.52
**2 h**	0.99	0.87	0.93	1.05
**2 h**	1.14	0.8	0.46	1.02
**2 h**	0.93	0.8	0.71	0.83

UL30 gene relative mRNA level

group	Res (0 µg/ml)	Res (3.75 µg/ml)	Res (7.5 µg/ml)	Res (15 µg/ml)
**1 h**	0.97	0.8	0.86	0.77
**1 h**	1.21	0.92	0.92	0.85
**1 h**	1.09	0.76	0.98	0.82
**1.5 h**	1.08	0.65	1	0.87
**1.5 h**	0.96	0.68	0.92	1.1
**1.5 h**	1.05	0.66	0.9	0.68
**2 h**	0.96	0.65	0.91	0.72
**2 h**	1	0.84	1.02	0.87
**2 h**	1.19	0.8	1.11	0.73

UL42 Relative mRNA level

group	Res (0 µg/ml)	Res (3.75 µg/ml)	Res (7.5 µg/ml)	Res (15 µg/ml)
**1 h**	0.99	0.94	1.01	0.68
**1 h**	1.01	0.89	0.96	0.73
**1 h**	1.18	0.84	0.95	0.82
**1.5 h**	1.05	0.84	0.88	0.86
**1.5 h**	0.95	0.9	0.85	0.85
**1.5 h**	1	0.97	0.86	0.85
**2 h**	0.97	0.54	0.85	0.72
**2 h**	0.96	0.64	0.86	0.69
**2 h**	0.84	0.6	0.84	0.82
