# Supplementary material for: Resveratrol Inhibits Pseudorabies Virus Replication by Targeting IE180 Protein
Source: Front Microbiol. 2022 Jun 2;13:891978. doi: 10.3389/fmicb.2022.891978 (PMC9203040; doi:10.3389/fmicb.2022.891978)
Supplement: Supplementary file 2 [file Data_Sheet_2.ZIP › Raw Data/Figure 3B (data sheet).pdf]

Cell Flourescence

**group**	**Res (0 µg/ml)**	**Res (15 µg/ml)**
1	22.19	4.72
2	20.78	3.52
3	22.26	3.35
