# Supplementary material for: Resveratrol Inhibits Pseudorabies Virus Replication by Targeting IE180 Protein
Source: Front Microbiol. 2022 Jun 2;13:891978. doi: 10.3389/fmicb.2022.891978 (PMC9203040; doi:10.3389/fmicb.2022.891978)
Supplement: Supplementary file 2 [file Data_Sheet_2.ZIP › Raw Data/Figure 4 (data sheet).pdf]

Relative activity luciferase

group	control	Res (0 µg/ml)	Res (3.75 µg/ml)	Res (7.5 µg/ml)	Res (15 µg/ml)
1	0.468168	13.64967	11.8107	4.673123	1.573854
2	0.491285	13.30139	10.96241	4.282738	0.981101
3	0.572006	14.92778	10.40602	4.952206	1.245554
