# Supplementary material for: Resveratrol Inhibits Pseudorabies Virus Replication by Targeting IE180 Protein
Source: Front Microbiol. 2022 Jun 2;13:891978. doi: 10.3389/fmicb.2022.891978 (PMC9203040; doi:10.3389/fmicb.2022.891978)
Supplement: Supplementary file 2 [file Data_Sheet_2.ZIP › Raw Data/Figure 5 (data sheet).pdf]

EPO gene relative mRNA level

group	pcDNA3.1 (+)	pIE180	Res+pcDNA3.1(+)
2 h	1	0.95	0.65
2 h	1	0.8	0.88
2 h	1	1.11	0.5
4 h	1	0.97	0.57
4 h	1	0.76	0.89
4 h	1	1.28	1.22
6 h	1	0.73	0.11
6 h	1	1.29	0.15
6 h	1	1.43	0.2
8 h	1	1.92	0.47
8 h	1	2.03	0.54
8 h	1	2.15	0.47

US1 gene relative mRNA level

group	pcDNA3.1 (+)	pIE180	Res+pcDNA3.1(+)
2 h	1	1.14	0.72
2 h	1	1.06	0.83
2 h	1	0.92	0.8
4 h	1	0.97	1.04
4 h	1	1.46	0.52
4 h	1	1.78	1.11
6 h	1	1.27	0.92
6 h	1	1.73	0.47
6 h	1	1.51	0.46
8 h	1	1.95	0.58
8 h	1	1.13	0.52

**8 h**	1	1.54	0.57

UL54 gene relative mRNA level

**group**	**pcDNA3.1 (+)**	**pIE180**	**Res+pcDNA3.1(+)**
**2 h**	1	1.14	0.67
**2 h**	1	1.13	0.8
**2 h**	1	1.14	0.69
**4 h**	1	1.39	0.81
**4 h**	1	0.71	0.87
**4 h**	1	1.44	0.99
**6 h**	1	1.23	0.12
**6 h**	1	1.39	0.19
**6 h**	1	0.77	0.15
**8 h**	1	1.61	0.26
**8 h**	1	0.92	0.21
**8 h**	1	1.22	0.19

Res+pIE180
0.31
0.68
1.26
0.88
1.09
1
0.12
0.16
0.09
0.23
0.21
0.25

Res+pIE180
0.53
0.52
0.7
0.95
0.77
0.8
0.13
0.09
0.11
0.32
0.67

0.36

Res+pIE180
0.6
0.57
0.66
0.71
0.64
0.54
0.1
0.05
0.07
0.15
0.13
0.11
