# Supplementary material for: Resveratrol Inhibits Pseudorabies Virus Replication by Targeting IE180 Protein
Source: Front Microbiol. 2022 Jun 2;13:891978. doi: 10.3389/fmicb.2022.891978 (PMC9203040; doi:10.3389/fmicb.2022.891978)
Supplement: Supplementary file 2 [file Data_Sheet_2.ZIP › Raw Data/Figure 7 (data sheet).pdf]

group	control	pIE180	$pIE180^{Thr601Ala}$	$pIE180^{Ser603Ala}$
1	5.21	21.66	20.01	21.51
2	4.86	20.69	20.35	21.73
3	5.72	20.75	19.92	22.16

Relative activity luciferase

pIE180^{Pro606Ala}	Res-pIE180	Res-pIE180^{Thr601Ala}	Res-pIE180^{Ser603Ala}
21.64	0.68	20.91	23.42
22.84	0.74	20.95	21.67
19.12	0.54	20.37	20.19

Res-pIE180^{Pro606Ala}
22.94
20.31
19.21
