# Supplementary material for: Resveratrol Inhibits Pseudorabies Virus Replication by Targeting IE180 Protein
Source: Front Microbiol. 2022 Jun 2;13:891978. doi: 10.3389/fmicb.2022.891978 (PMC9203040; doi:10.3389/fmicb.2022.891978)
Supplement: Supplementary file 2 [file Data_Sheet_2.ZIP › Raw Data/Figure 8 (data sheet).pdf]

EPO gene relative expr

group	pcDNA3.1(+)	pIE180	pIE180^{Thr601Ala}	pIE180^{Ser603Ala}	pIE180^{Pro606Ala}
**2 h**	1.23	1.84	2.63	2.37	2.11
**2 h**	0.66	1.48	2.86	3.02	2.25
**2 h**	1.12	1.16	2.65	1.39	2.3
**4 h**	1.11	2.49	1.28	1.33	2.53
**4 h**	0.75	1.77	1.35	2.17	2.11
**4 h**	1.14	1.6	1.4	2.81	2.38
**6 h**	0.8	2.27	1.46	3.11	1.74
**6 h**	1.17	2.32	1.7	3.24	1.69
**6 h**	1.03	2.35	1.68	3.13	1.45
**8 h**	1.31	1.89	1.86	1.41	0.92
**8 h**	0.74	2.01	2	1.66	1.15
**8 h**	0.95	1.68	1.91	1.49	1.54

US1 gene relative expr

group	pcDNA3.1(+)	pIE180	pIE180^{Thr601Ala}	pIE180^{Ser603Ala}	pIE180^{Pro606Ala}
**2 h**	0.71	2.85	3.21	5.07	2.81
**2 h**	1.09	3.81	3.32	3.89	2.13
**2 h**	1.19	3.21	3.59	3.79	2.2
**4 h**	1.05	4.94	2.94	5.25	2.62
**4 h**	1.21	4.76	2.84	6.34	3.66
**4 h**	0.75	4.92	2.97	5.68	3.32
**6 h**	1.02	6.9	3.71	4.05	2.05
**6 h**	0.86	6.91	3.8	3.98	1.97
**6 h**	1.12	6.89	3.81	4.6	1.64
**8 h**	0.65	1.46	2.88	1.47	0.94
**8 h**	1.33	1.6	2.82	1.33	0.97
**8 h**	1.02	1.2	2.45	1.3	1.21

UL54 gene relative expr

group	pcDNA3.1(+)	pIE180	pIE180^{Thr601Ala}	pIE180^{Ser603Ala}	pIE180^{Pro606Ala}
**2 h**	1.13	1.76	5.89	6.78	1.19
**2 h**	0.82	1.58	5.53	4.05	1.46
**2 h**	1.05	1.01	6.1	3.15	1.48
**4 h**	1.32	0.9	4.8	3.2	1.99
**4 h**	0.74	0.67	4.59	3.25	2.21
**4 h**	0.94	0.59	4.48	3.78	1.15
**6 h**	0.94	0.14	4.18	4.27	1.68
**6 h**	0.77	0.18	4.27	4.12	1.6
**6 h**	1.29	0.1	4.13	4.01	1.56
**8 h**	0.7	1.3	3.46	0.81	1.67
**8 h**	0.92	1.01	3.08	1.01	1.32
**8 h**	1.38	0.78	2.87	1.09	0.79

ession level

Res-pIE180	Res-pIE180^{Thr601Ala}	Res-pIE180^{Ser603Ala}	Res-pIE180^{Pro606Ala}
2.23	3.21	3.02	1.72
2.04	2.64	2.7	1.88
2.31	3.03	3.25	1.85
3.7	3.37	2.39	3.16
4.19	2.68	4.66	1.66
3.33	3.14	2.89	3.12
1.28	4.56	3.1	1.82
1.39	4.66	2.9	2.1
1.27	4.55	3.39	1.97
0.6	2.36	2.96	1.99
0.44	2.25	2.48	2.27
0.52	2.3	2.33	2.67

ession level

Res-pIE180	Res-pIE180^{Thr601Ala}	Res-pIE180^{Ser603Ala}	Res-pIE180^{Pro606Ala}
0.74	3.6	3.79	4.6
1.08	4.6	3.72	4.04
0.71	3.57	4.54	4.41
0.51	1.55	4.11	4.08
0.36	1.47	5.12	3.82
0.54	1.35	5.29	3.84
0.4	1.66	2.46	2.27
0.27	1.58	2.33	2.45
0.29	1.62	2.44	2.45
0.43	2.72	1.96	1.68
0.29	2.05	1.36	1.8
0.39	1.79	1.42	2.29

ression level

Res-pIE180	Res-pIE180^{Thr601Ala}	Res-pIE180^{Ser603Ala}	Res-pIE180^{Pro606Ala}
1.71	2.41	3.88	2.15
1.95	2.26	3.36	2.79
1.83	2.59	4.14	2.33
1.17	2.57	5.64	3.18
0.87	1.65	5.27	3.5
1.02	1.74	5.35	3.87
1.44	2.43	4.14	4.4
1.21	2.15	3.94	4.11
1.24	2.37	4.43	3.84
0.41	1.19	3.42	2.82
0.36	1.4	2.21	2.55
0.4	0.99	3.11	2.53
