# Supplementary material for: Resveratrol Inhibits Pseudorabies Virus Replication by Targeting IE180 Protein
Source: Front Microbiol. 2022 Jun 2;13:891978. doi: 10.3389/fmicb.2022.891978 (PMC9203040; doi:10.3389/fmicb.2022.891978)
Supplement: Supplementary file 2 [file Data_Sheet_2.ZIP › Raw Data/Figure 2 (data sheet and images blot).zip!/Figure 2 (data sheet and images blot)/Figure 2 (data sheet).pdf]

Expression test of IE180 protein PRV infection

group	Res(0μg/ml)	Res (15μg/ml)
4 h	0.38	0.46
4 h	0.37	0.34
4 h	0.39	0.4
6 h	0.43	0.39
6 h	0.37	0.38
6 h	0.4	0.37
8 h	0.3	0.38
8 h	0.35	0.39
8 h	0.32	0.37

Over-expression test of IE180 protein

group	Res(0μg/ml)	Res (15μg/ml)
4 h	0.36	0.33
4 h	0.35	0.42
4 h	0.35	0.31
6 h	0.3	0.35
6 h	0.33	0.34
6 h	0.35	0.35
8 h	0.29	0.36
8 h	0.33	0.35
8 h	0.34	0.35
